# An improved approach to estimating the infiltration characteristics in surface irrigation systems

**DOI:** 10.1371/journal.pone.0234480

**Published:** 2020-06-15

**Authors:** Mohamed Khaled Salahou, Xiyun Jiao, Haishen Lü, Weihua Guo

**Affiliations:** 1 State Key Laboratory of Hydrology–Water Resources and Hydraulic Engineering, Hohai University, Nanjing, China; 2 College of Agricultural Engineering, Hohai University, Nanjing, China; Hellenic Agricultural Organization - Demeter, GREECE

## Abstract

The most common methods for estimating the infiltration function are measurements through a double-ring infiltrometer (DRI) and empirical models. Infiltration data always exhibit different kinds of scatter, which affect the accuracy of the estimated infiltration function. This study presents a new methodology to calibrate the infiltration function. The suggested approach is based on combining the DRI method with the changes in the measured soil water content. Furrow irrigation experiments were conducted to estimate the infiltration function using different methods and to investigate the effect of data scatter on the reliability of the estimated infiltration function. Furrow elevations were observed, and for each irrigation event advance times, recession times, and inflow rates were observed. The infiltration depths were measured as a function of the change in the soil water content before and after irrigation event. Infiltration parameters were estimated using DRI treatment, empirical model (Kostiakov model), and suggested approach. Measured and simulated infiltration depths using the described methods were compared. The results show that the infiltration depths estimated using a DRI were lower than the observed infiltration depths, while the infiltration depths estimated using the empirical model were higher than the observed infiltration depths. The results indicate that the infiltration function estimated using the recommended approach was more accurate and reasonable than the infiltration function estimated using the DRI, and empirical (Kostiakov model) methods. In addition, the proposed approach can reduce the required measurements during the irrigation event, and can also reduce the potential scatter in the estimated infiltration function that results from soil variability and measurement errors.

## 1. Introduction

Soil infiltration characteristics are important for the evaluation, design and management of surface irrigation systems. Empirical infiltration models and double-ring infiltrometer (DRI) methods are widely used to estimate the infiltration function in surface irrigation applications. The required data for infiltration determination collected during an irrigation event (i.e., advance and recession times, inflow rate, and slope, which are used to describe many approaches in surface irrigation) or prior to an irrigation event form the array of existing field measurements (e.g., using a DRI). The advance and recession times have been defined in many studies e.g., [1–4], where the advance time is the time-distance relationship in water front advance, and the recession time is the time-distance relationship begins when the depth of surface water at the upstream decreases to zero and continues until the surface is drained.

Empirical infiltration models differ depending on computational approaches and data requirements. Many studies have been conducted to define coefficients in empirical infiltration models based on the volume balance approach (i.g., the two-point method [5–10], one-point method [11–13], Saint-Venant approach [14, 15], zero-inertia approach [16–18], and kinematic-wave approach [18, 19]). These approaches depend on different levels of measurements, and errors in the measurement data can cause substantial errors in assessing the soil infiltration function [20]. Hence, when computational approaches that depend on limited measurement data (i.e., the one-point and two-point methods) are used to estimate the infiltration function, the results are not always reliable. Bautista et al. [21] concluded that the two-point method is inherently uncertain because of the sensitivity of the calculations to uncertain advance data and the limited infiltration information provided by the advance phase. The unreliable results of estimation infiltration parameters can occur not only with the two-point method, but also with any method that relies solely on advance data (measurement of the water advance time to a known distance) [22]. Even when the observations are collected carefully, the results can be very sensitive to the location of observation stations [7, 23]. In addition, the methods that depend on limited measurement data can be restrictive for field use in many situations [24, 25]. Conversely, the accuracy of the estimated infiltration function increases with computational approaches that depend on large amounts of measurement data. However, in this case, the collection of a large amount of data (i.g., observations of the advance time, recession time, and soil water content every 5 m along the field length instead of at one-point or at two-points) can decrease the data accuracy, particularly for large-scale projects when using laborers with imperfect knowledge about data collection [26–28].

Recently, studies have been performed to calibrate the infiltration parameters of empirical infiltration models with the aid of hydrodynamic simulation software, such as WinSRFR [3, 4] (e.g., [29–32]) and SISCO [33] (e.g., [34–36]), which offer sufficient tools for implementing the previous computational approaches.

Other studies have estimated the infiltration function in field situations using small area methods. The small area methods involve the use of blocked-furrow infiltrometers [37], bypass infiltrometers [38], the inflow-outflow method [39], flowing infiltrometers [40], flow-through infiltrometers [41], a single ring infiltrometer [42] and a DRI [1, 2, 43]. The DRI instruments are very easy to use and have been used by several researchers in China [8]. When a ring infiltrometer or DRI is used, the saturated soil hydraulic conductivity is assumed to be equal to the final infiltration rate [44] or 0.667 [45]. In addition, the soil is assumed to be homogeneous within the measured area. However, the soil heterogeneity and soil spatial variability influence the measurements. In particular, the measurements made using the ring infiltrometers and DRIs may give size-dependent results. Many studies have investigated the effects of the heterogeneity on the infiltration rate, and the main conclusion of these studies is that the mean infiltration rate depends on the measurement size in heterogeneous media (the mean infiltration rate increases with increasing measurement size up to a point, above which the infiltration rate becomes constant) [46–48], the degree of heterogeneity in the media [46], the intrinsic structure of the heterogeneous media and the flow process [49], and the location and sampling size (soil contact area of the dish infiltrometer) [50]. Furthermore, even in homogeneous media, the mean infiltration rate increases with increasing measurement size [51].

In previous methods, the reliability of the estimated infiltration parameters depends on the selected approach, which is basically dependent on the amount, variety and accuracy of the available measurement data, and measurement errors can cause substantial errors in estimating the infiltration function. In addition, the parameters in empirical infiltration models have no physical relevance and do not generally consider specific initial and boundary conditions, and the small area methods fail to provide infiltration characteristics representative of the true overall field values. Therefore, an improved approach to estimating the infiltration characteristics in irrigation systems is needed to decrease the number of observations and increase the accurate of the estimated infiltration function. Hence, the objectives of this study were as follows: (1) to propose a new methodology to estimate infiltration characteristics in surface irrigation systems; (2) to evaluate infiltration functions estimated using an empirical infiltration model (the Kostiakov equation in this study), a DRI and the proposed method; (3) to investigate the effect of the selected approach and data scatter on the estimated infiltration function (the measured and simulated cumulative infiltration depths were compared, where the cumulative infiltration depths were observed based on soil water content measurements).

## 2. Materials and methods

### 2.1. Experimental area

Field experiments were conducted at the Wuqiao Agricultural Experimental Station in Wuqiao (116°37'E, 37°65'N), Hebei Province, China. Wuqiao County is a typical area of intensive agriculture in the North China Plain. Cotton is an important crop to be cultivated in the study area, which is planted in furrow irrigation systems with growing periods total more than 160 days from the middle of April to the middle of October. The climate of the region is a continental monsoon climate, with an average annual evaporation range of 1500–1800 mm. The mean annual precipitation and temperature at the study site are approximately 576 mm and 12.6°C, respectively. Rainfall generally occurs during the summer. Particle size distribution (i.e., soil texture) and bulk density of the soil at the site were determined according to Klute [52]. The soil texture was determined every 10 cm to a depth of 100 cm at three locations. The soil of the studied area is a silty loam soil (61.9±5% silt, 28.3±7.4% sand, and 9.8±2.5% clay on average ± standard deviation) with an average bulk density of 1.43 g cm^-3^ for the 50 cm soil depth.

### 2.2. Experimental treatments

**Fig 1:**
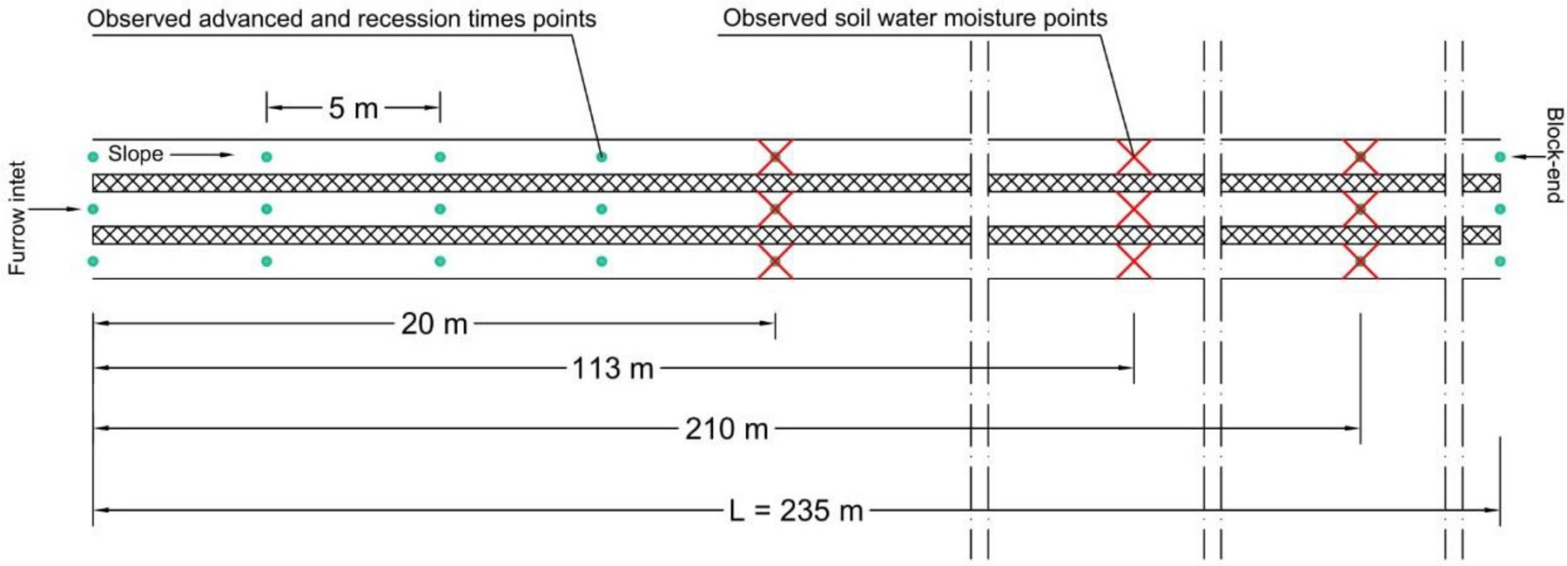
A plan view showing a general layout, observed advanced and recession times points, and observed soil water moisture points.

A total of 12 furrow irrigation assessments were performed in the study area. The furrows had the following average characteristics: V-shaped, 0.4 m top width, 0.2 m maximum height, 235 m length, and 0.6 m furrow space. Furrow elevations were observed using an optical level, and for each irrigation event, the advance times, recession times (every 5 m along the furrow length) (Fig 1), and inflow rates (measured using a typical water flow meter) were observed. All furrows slope in the downstream direction, with an average slope of between 0.0006 and 0.0009 m m^-1^ (correlation coefficient of the linear regression analysis of the slopes is between 0.70 and 0.92). It was confirmed that only one furrow was irrigated at a time, that the full amount of inflow through the service point was applied to that furrow, and that the buffering capacity of the farm channel removed most of the short-term variation. Consequently, the time-averaged inflow rate was used in all the evaluations.

The soil samples were taken with a soil auger at 20, 113, and 210 m from the furrow head (Fig 1) in each furrow over 0–100 cm in depth at 20 cm intervals before irrigation events and one day after irrigation events for the soil water content measurements. The soil water content was determined by gravimetric analysis to assess the change in the soil water status prior to and after irrigation event using the oven drying method with 10 cm soil layers down to 100 cm at 20, 113, and 210 m in the longitudinal direction of the furrows.

The advance and recession times of furrow 12 were not obtained near the furrow downstream due to the field conditions. Therefore, the evaluation of furrow 12 was not included in the analysis.

### 2.3. Infiltration function

The infiltration functions were determined using the following methods:

#### 2.3.1. Double-ring infiltrometer

Some of the analyses presented herein are based on the use of a DRI to estimate the infiltration function. The DRI measurements were repeated at three different locations in the study area. The ring diameters used in this study were 30 and 60 cm. Detailed descriptions of the double-ring infiltrometer were provided by [44].

#### 2.3.2. Kostiakov model

The analysis presented herein also uses the simplest and most common approximations of an empirical model for estimating infiltration, which is described by the Kostiakov equation [53]. The Kostiakov equation can be written as

$$Z=k\tau^{\alpha }$$ (1)

where *Z* is the cumulative infiltration depth in mm, *τ* is the intake opportunity time in hours (the difference between advance and recession time), *k* is a coefficient with units of mm hr^-α^, and *α* is an empirical constant (*k* > 0, and 0 < *α* <1).

The simple post-irrigation volume balance (PIVB) [54–56] was used to solve the Kostiakov equation parameters (*k*, *α*). WinSRFR software [3, 4, 57] provides a hydraulic simulation tool for solving the PIVB. The input data for WinSRFR software for each furrow irrigation event included the irrigation water requirement, system geometry (furrow length, space, and cross section), average furrow slope, roughness coefficient (in this study, *n* = 0.04 [21]), inflow rate, cut-off time, downstream conditions (in this study, blocked-ended furrow), and advance and recession times at each measurement distance within each furrow (in this study, every 5 m along the furrow length). In addition, because the Kostiakov formula was selected as an empirical infiltration function to estimate the infiltration function, the *α* parameter was also entered as input. The simulated advance and recession times were compared with the observed times, and if the fit was poor, then a new value was assigned to the *α* parameter. This procedure was repeated until a good fit between the simulation and observation data was achieved. In this manner, the values of *α* and the *k* parameter were obtained.

#### 2.3.3. Suggested approach

As mentioned, when a DRI is used, the soil is assumed to be homogeneous within the measured area, and the measurements made using the DRIs may give size-dependent results. On the other hand, the parameters in Kostiakov infiltration models have no physical relevance and do not generally consider specific initial and boundary conditions, and the accuracy of the estimated infiltration function increases with large amounts of measurement data. In the suggested approach, the soil moisture data before and after irrigation used to estimate the parameters of the Kostiakov equation:

$$Z_{i}=Z_{C}$$ (2)

where *Z*_*i*_ is the cumulative infiltration depth as given in Eq (1), and *Z*_*c*_ is the cumulative infiltration depth estimated from the soil water content described as follows:

$$Z_{C}=\int_{0}^{D}\left[\theta \left(Z,t\right)-\theta \left(Z,0\right)\right]dZ$$ (3)

where θ(*Z*, *t*) is the soil water content measured after the irrigation event (m^3^ m^-3^); θ(*Z*, 0) is the initial soil water content measured before the irrigation event at 20, 113, and 210 m from the furrow head in each furrow (m^3^ m^-3^); and D is the soil layer depth (cm). The soil depth was considered to be 100 cm.

By substituting Eqs (1) and (3) in (2), we obtained a new form of the volume balance equation that can be applied to the two points measured along the furrow:

$$k\tau_{1}^{\alpha }=\int_{0}^{D}[\theta_{1}\left(Z,t\right)-\theta_{1}\left(Z,0\right)]dZ$$ (4)

$$k\tau_{2}^{\alpha }=\int_{0}^{D}[\theta_{2}\left(Z,t\right)-\theta_{2}\left(Z,0\right)]dZ$$ (5)

Then, the values of *k* and *α* are obtained by solving the system formed by Eqs (4) and (5):

$$\alpha =\frac{Ln\frac{\int_{0}^{D}[\theta_{1}\left(Z,t\right)-\theta_{1}\left(Z,0\right)]dZ}{\int_{0}^{D}[\theta_{2}\left(Z,t\right)-\theta_{2}\left(Z,0\right)]dZ}}{ln\frac{\tau_{1}}{\tau_{2}}}$$ (6)

$$k=\frac{\int_{0}^{D}[\theta_{1}\left(Z,t\right)-\theta_{1}\left(Z,0\right)]dZ}{\tau_{1}^{\alpha }}$$ (7)

As noted above, the soil water content was measured at 20, 113, 210 m from the furrow head in each furrow before and after irrigation events. The opportunity times on the right-hand side of Eqs (6) and (7) were calculated from the difference between the advance and recession times during the irrigation events at the same previous points (20, 113, 210 m along the furrow length). Since the opportunity times were obtained during the irrigation events and the soil moisture changes were observed before and after irrigation events, the evaporation losses should be accounted for. In this study, the evaporation losses were ignored and to reduce the effect of the evaporation losses on the results, the measured points were covered by plastic mulch. In addition, the result of Eq (6), for certain practical cases, e.g., *τ*_2_ > *τ*_1_, could exceed the recommended range of the *α* parameter (0 < *α* <1). To solve this problem, a new procedure is suggested that depends on the combination of the DRI method with the changes in the measured soil water content. The details about this procedure are illustrated in detail in the Results and Discussion section.

### 2.4. Infiltration model performance

The estimated infiltration models efficiency were evaluated by the root mean square errors (RMSE), the mean absolute errors (MAE), and the mean relative errors (MRE). The RMSE provides an indication of how well the simulated values match the observed values, and has a minimum value of zero. The MAE tells how big of an error can expect from the estimation on average, and measures the average magnitude of the errors in a set of estimations, without considering their direction. The MRE is a measure of prediction accuracy of an estimating method in statistics. RMSE, MAE, and MRE can be written as:

$$RMSE=\sqrt{\frac{1}{N}\overset{N}{\underset{i=1}{\sum }}{\left(Z_{S}-Z_{O}\right)}_{i}^{2}}$$ (8)

$$MAE=\sqrt{\frac{1}{N}\overset{N}{\underset{i=1}{\sum }}{\left|Z_{S}-Z_{O}\right|}_{i}}$$ (9)

$$MRE=\frac{1}{N}\overset{N}{\underset{i=1}{\sum }}{\left|\frac{Z_{S}-Z_{O}}{Z_{O}}\right|}_{i}$$ (10)

where N is the number of observations, Z_S_ is the simulated infiltration depth, and Z_O_ is the observed infiltration depth.

## 3. Results and discussions

### 3.1. Double-ring infiltrometer

**Fig 2:**
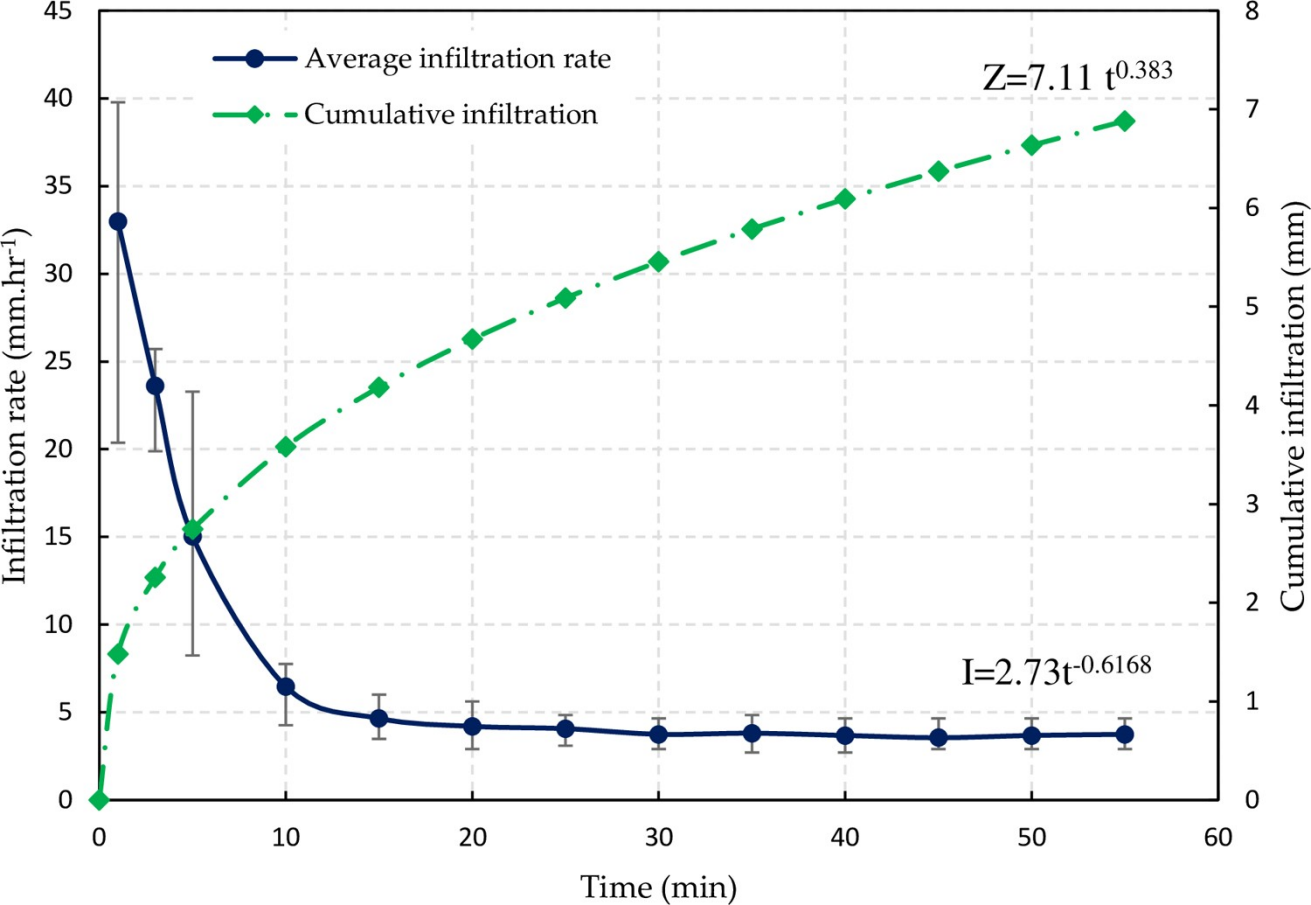
Field infiltration rate, I (mm hr^-1^) and cumulative infiltration depth Z (mm), the vertical bars represent the maximum and minimum value at each point.

The measured intake rates for the individual infiltration runs were obtained at 2–5 minute intervals at each of the three sites in the study area. The maximum, minimum, and average points are shown in the figure at regular intervals (**Fig 2**). The infiltration rate (I, mm hr^-1^) fitted to the Kostiakov equation was found in the experimental field as I = 2.73t^-0.6168^.

The cumulative infiltration depth *Z* in millimeters was integrated from the infiltration rate function and reported as Z = 7.11t^0.383^, where *Z* was given in millimeters and t was given in hours.

In the border irrigation system, the cumulative infiltration depth can be used directly. However, in the furrow irrigation system, the parameter *k* can be converted so that it is compatible with the furrow system by dividing by the furrow spacing. Then, the cumulative infiltration depth for the furrow system can be reported as follows: Z = 11.86t^0.383^, where *Z* is given in millimeters, and t is given in hours.

### 3.2. Furrow irrigation events

Table 1 provides details of the average slope, inflow rate, inflow duration, final advance time and infiltrated water volume of the furrows.

**Table 1:** Furrow observation data.

Furrow no.	Slope (m m^-1^)		Inflow rate (l s^-1^)		Flow duration (min)	Final advance time (min)	Infiltrated volume (m^3^)
	Average slope	σ_slope_	Average inflow	σ_q_			
1	0.0009	0.0640	1.14	0.11	115.4	119.50	7.89
2	0.0008	0.0592	1.39	0.09	115.4	123.32	9.62
3	0.0007	0.0549	1.14	0.68	100.5	95.42	6.87
4	0.0008	0.0582	1.18	0.16	96.0	102.55	6.80
5	0.0007	0.0521	1.18	0.09	115.3	122.00	8.16
6	0.0006	0.0481	1.04	0.43	106.1	119.50	6.62
7	0.0009	0.0618	1.17	0.27	104.9	114.22	7.36
8	0.0008	0.0554	1.49	0.50	67.0	75.37	5.99
9	0.0008	0.0554	1.40	0.26	104.6	117.62	8.78
10	0.0009	0.0694	1.10	0.30	104.9	108.77	6.93
11	0.0009	0.0678	1.08	0.31	92.1	100.33	5.97
12	0.0008	0.0640	1.18	0.11	104.8	-	7.42

The parameters of the Kostiakov equation (*k*, *α*) were estimated using WinSRFR software. The simulated advance curve was adjusted by modifying the *α* parameter, and the initial value of the *α* parameter was set as equal to the value estimated from the DRI (*α* = 0.383). Then, the k parameter was estimated. The simulated and observed advance curves had a good fit (Table 2) when the previously described α parameter value was used, which means that the *α* parameter estimated from the DRI can be used directly for estimating the k parameter value.

**Table 2:** RMSE of advance and recession times.

Furrow No	1	2	3	4	5	6	7	8	9	10	11	12	RMSE ave.
RMSE advance (min)	1.69	4.91	2.49	6.63	3.48	2.1	5.17	1.80	3.69	2.23	4.2	-	3.49
RMSE recession (min)	54.7	46.2	56.4	57.6	34.2	48.2	15.3	28.6	20.2	24.4	35.3	-	38.28

The *k* parameter values estimated directly from DRI-derived *α* parameters are useful for testing the accuracy of different roughness coefficient (*n*) values, which are difficult to evaluate with blocked-end systems. In the surface irrigation design, initial values of the *α* and *n* parameters were set for solving the Kostiakov equation using the simple post-irrigation volume method (which was solved using WinSRFR software). However, in this study, the *α* parameter was estimated directly from the DRI; therefore, in this case, the only unknown parameter is *n*. Then, different values of the roughness coefficient from the range (0.04–0.16) recommended by Anwar et al. [58] were evaluated by comparing the observed and simulated data for each irrigation event. The roughness coefficient (*n* = 0.04) achieved the best fit between the observed and simulated advance and recession times for all irrigation events.

**Fig 3:**
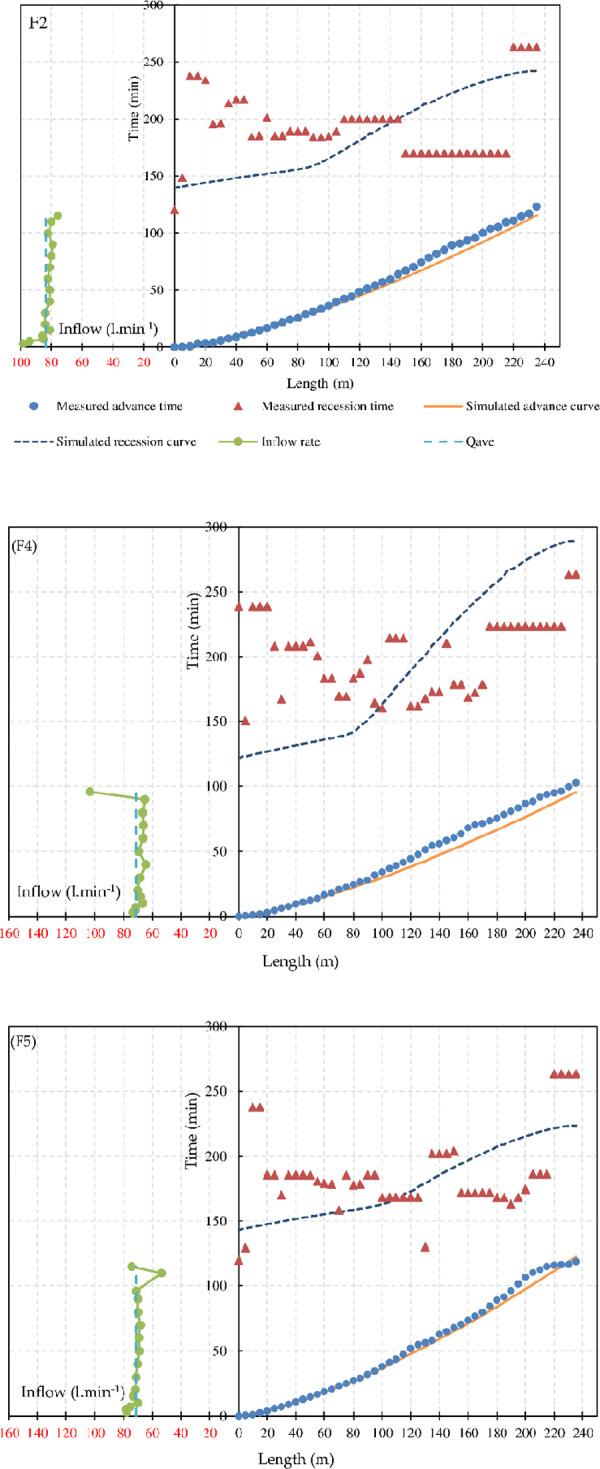
Measured (dots) and simulated (lines) advance and recession curves, inflow rates, average inflow rates, and ordinary least squares regression equations of F2, F4, and F5.

Table 2 shows that the modeling of the advance phase of the irrigation events was reasonable (average RMSE of advance times = 3.49 min), but the results for the recession were not as good, with an average RMSE of 38.28 min. These results were obtained because it is easy to measure the advance time accurately and reliably in surface irrigation events. Conversely, the recession times are difficult to obtain; therefore, the measurement data scatter of the recession times was expected to be large (Fig 3).

From the field data, the field bottom elevation was mildly irregular (Fig 3). This irregularity causes variations in recession times, as the measurements suggest. However, with the given slope variations, the recession times are expected to mostly increase with distance down the field. In other words, the variations in recession time seem extreme for the variations in field elevation. At the same time, the recession measurements seem to be consistent among tests. Thus, it is difficult to determine the cause of these recession time variations. The possible reasons are the slope variations (Table 1) at some parts of the furrows, measurement errors, and very large variations in soil texture in the fields. However, the soil texture characteristics along the field were tested at only three locations.

Infiltration data, whether measured with a DRI or with an entire furrow using an infiltrometer, always exhibit scatter. It is possible that the scatter is the result of soil variability and measurement errors. We must measure the potential scatter of these data and use our knowledge about infiltration (and roughness) variability to develop design/operational recommendations. Therefore, we are interested in finding solutions that will be robust, i.e., that will be the least affected if actual conditions differ from those assumed in the simulation design, which may not be the solution with the best distribution uniformity and application efficiency.

To address the data scatter problem, Kostiakov equation parameters were estimated using the soil water content measurements (Eqs (6) and (7)). However, the results of Eq (6) can extend beyond the recommended *α* parameter range. At the same time, the *α* parameter, which was estimated with a DRI, is accurate. Therefore, the use of *α* = 0.383 (instead of estimating the *α* parameter from Eq (6)) were used for the estimation of the *k* parameter from Eq (7). Eq (7) can be written for the soil water content observation location i as follows:

$$k_{i}=\frac{\int_{0}^{D}\theta_{i}\left(Z,t\right)-\theta_{i}\left(Z,0\right)dZ}{\tau_{i}^{0.383}}$$ (11)

The average *k* parameter can be obtained as follows:

$$k=\frac{1}{n}\overset{i=n}{\underset{i=1}{\sum }}\frac{\int_{0}^{D}\theta_{i}\left(Z,t\right)-\theta_{i}\left(Z,0\right)dZ}{\tau_{i}^{0.383}}$$ (12)

where n is the number of observation locations of soil water content.

**Fig 4:**
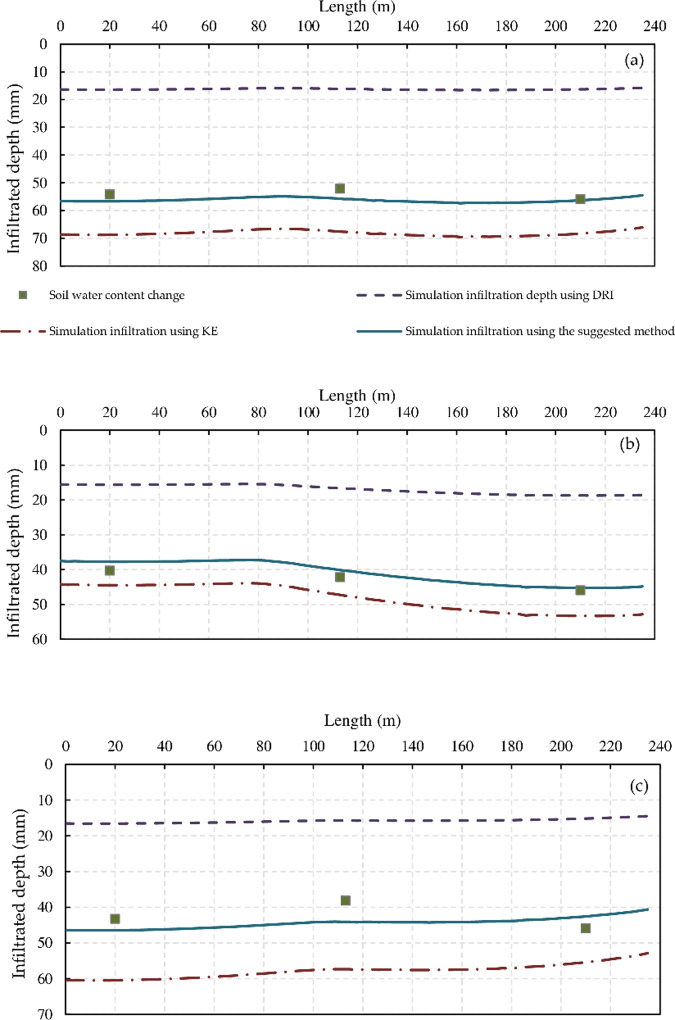
Accumulative infiltration depth obtained from double-ring infiltrometer (DRI), Kostiakov equation (KE), and suggested method of F2, F4, and F5.

The infiltration function estimated from the suggested method (*α* derived from the DRI and the *k* parameter obtained by Eq (12)) was more accurate and reasonable (**Table 3**) than that estimated by other methods (the DRI and Kostiakov equation, which use observed advance and recession times) (Fig 4).

**Table 3:** Infiltration function parameters, RMSE, MAE, and MRE of the infiltration depth.

Furrow NO.	Infiltration parameters														
	Double-ring infiltrometer		RMSE	MAE	MRE	Kostiakov equation		RMSE	MAE	MRE	Suggestion approach		RMSE	MAE	MRE
	α	k^[^^1^^]^				α	k^[^^2^^]^				α	k^[^^3^^]^			
	-	mm.hr^-a^	mm	mm	%	-	mm.hr^-a^	mm	mm	%	-	mm.hr^-a^	mm	mm	%
F1	0.383	11.855	23.3	22.55	0.57	0.383	40.169	19.2	16.91	0.44	0.383	29.164	2.5	2.37	0.06
F2			37.8	37.79	0.70	0.383	49.667	14.2	14.11	0.26	0.383	40.953	1.3	1.16	0.02
F3			25.5	25.48	0.60	0.383	33.531	6.1	6.07	0.14	0.383	28.386	0.4	0.37	0.01
F4			25.8	25.76	0.60	0.383	33.727	5.7	5.56	0.13	0.383	28.620	0.9	0.85	0.02
F5			26.8	26.60	0.62	0.383	43.307	15.9	15.31	0.37	0.383	33.270	3.9	3.51	0.08
F6			24.9	24.72	0.60	0.383	33.68	8.9	8.21	0.20	0.383	30.466	0.5	0.44	0.01
F7			19.8	19.34	0.56	0.383	41.168	18.2	17.90	0.54	0.383	27.589	3.5	3.04	0.09
F8			24.2	24.16	0.61	0.383	32.386	3.3	2.80	0.07	0.383	29.917	1.5	1.38	0.03
F9			24.6	24.31	0.62	0.383	49.763	23.8	23.09	0.61	0.383	29.506	4.9	4.38	0.11
F10			27.4	27.33	0.64	0.383	37.901	7.1	6.53	0.16	0.383	34.498	2.7	2.46	0.06
F11			23.7	23.53	0.59	0.383	30.99	4.7	3.78	0.10	0.383	29.174	1.6	1.45	0.04
F12						-	-				-	-			
Average	0.383	11.855	25.8	25.60	0.61	0.383	38.754	11.6	10.93	0.27	0.383	31.049	2.2	1.95	0.05
Maximum	-	-	37.8	37.79	0.70	-	49.763	23.8	23.09	0.61	-	40.953	4.9	4.38	0.11
Minimum	-	-	19.8	19.34	0.56	-	30.990	3.3	2.80	0.07	-	27.589	0.4	0.37	0.01
Standard deviation	-	-	4.5	4.58	0.04	-	6.699	7.0	6.77	0.19	-	3.885	1.5	1.3	0.04

[1] Based on DRI treatment,

[2] Based on WinSRFR software and trial-and-error

[3] Based on Eq (12)

Fig 4 illustrates the infiltration depths obtained using the following methods: (i) DRI method (Z = 11.86t^0.383^, where t is the intake opportunity time); (ii) Kostiakov equation (KE)), where the parameters (*k*, *α*) of the KE equation were established with the aid of the WinSRFR and trial-and-error. As mentioned, the *α* parameter estimated from the DRI was achieved the best fit. However, the k parameter differs among the furrows; (iii) the proposed method (α derived from the DRI and the k parameter obtained by Eq (11) (**Table 3**). In addition, the soil water changes (Eq (3)) were presented at three location along the furrow.

The infiltration parameters obtained from the DRI, the Kostiakov equation, and the suggested method, along with the RMSE, MAE, and MRE of the cumulative infiltration depth, are summarized in **Table 3**.

The RMSE of the infiltration depth obtained by applying the suggested method ranged from 4.9 to 0.4 mm, while those obtained by the DRI and the Kostiakov equation ranged from 37.9 to 19.8 mm and 23.8 to 3.3 mm, respectively (**Table 3**). The MRE values of the infiltration depth obtained by applying the suggested approach were from 0.01 to 0.11%, while those obtained by the DRI and the Kostiakov equation ranged from 0.56 to 0.70% and 0.07 to 0.61%, respectively. The RMSE and MRE for furrow F10 was the lowest when the infiltration function was estimated using the suggested method, possibly because the soil water points used for observations were not sufficient to obtain a clear relation for this furrow. However, in all irrigation events, the RMSE, and MRE values of the infiltration depth obtained by applying the suggested method were the lowest, with an average of 2.20 mm and 0.05%, respectively.

**Fig 5:**
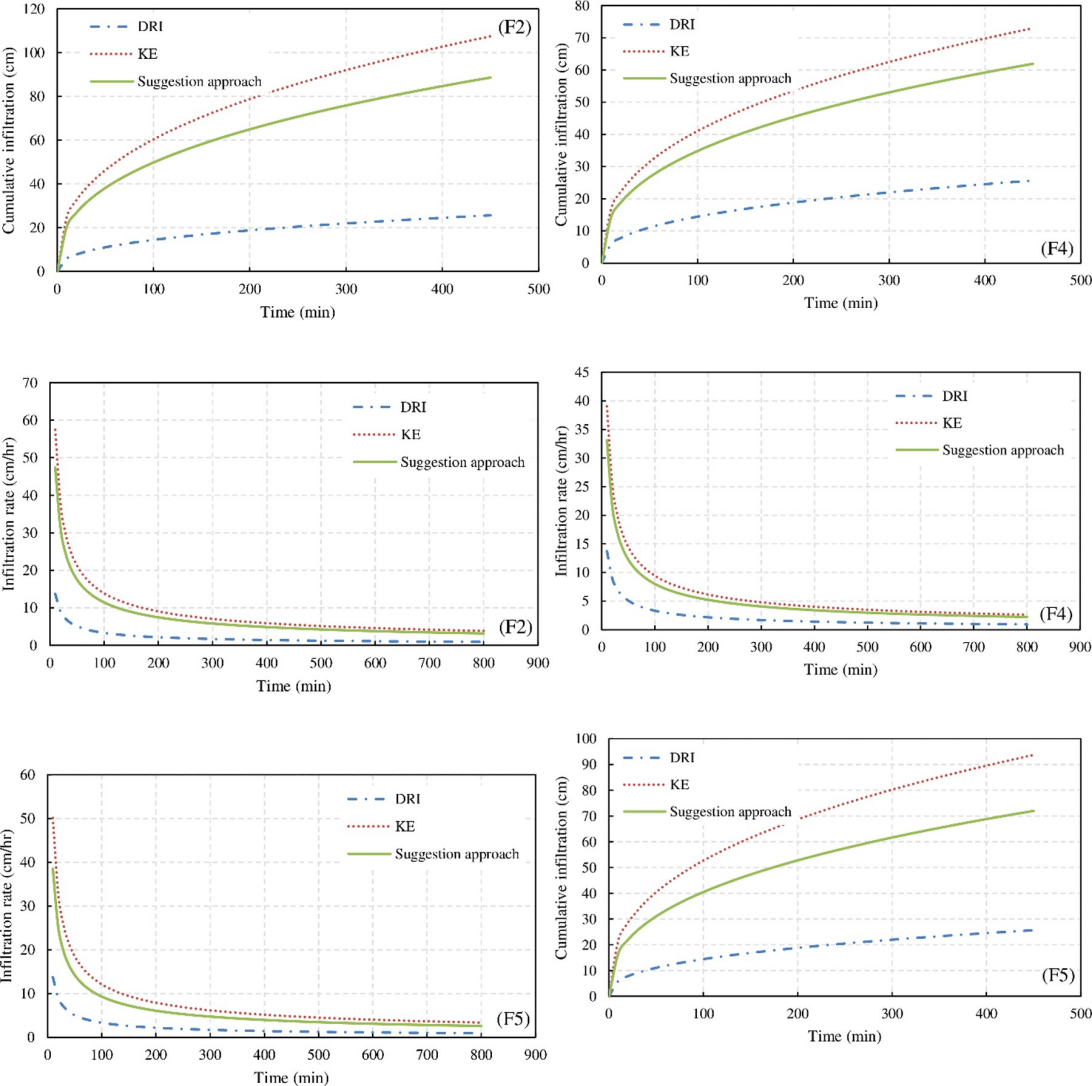
Infiltration rate and cumulative infiltration obtained from double-ring infiltrometer (DRI), Kostiakov equation (KE), and suggested approach of (F2) furrow 2, (F4) furrow 4, and (F5) furrow 5.

It is worth noting that when comparing the estimated infiltration functions, both cumulative infiltration and infiltration rates must be considered. A calibrated infiltration function may poorly predict cumulative infiltration but may accurately predict infiltration rates over long times. Likewise, a function may predict cumulative infiltration and infiltration rates at short times but may poorly predict long-term infiltration rates and therefore cumulative infiltration. The functions generated for all furrows predicted nearly the same long-term infiltration rates for the KE and the suggested functions, even if the cumulative infiltration values differed (Fig 5). Although the infiltration function which estimated from DRI treatment was underestimated, the estimated function provides information about the infiltration function shape in the field because the simulated and observed advance curves had a good fit when the DRI-derived *α* parameter value was used (the shape of the infiltration curves obtained from *a* parameter). The estimated infiltration function based on KE method required a large number of measurements (i.e., advance times, recession times, inflow rate, and slope), and the infiltration function accuracy increases with increasing measurement size (e.g., observations of the advance time, and recession time every 5 m along the field length instead of every 10 m). However, estimating the infiltration function based on the suggestion method can reduce the required measurements during the irrigation event, only advance and recession times are required which observed at three location along furrow length in this study. Furthermore, the suggested approach can reduce the number of unknown parameters (*a*, *n*), only the roughness coefficient (*n*), instead of both *α* and *n*, needs to be established with the PIVB method and through trial and error (calibrating both *a* and *n* parameters at the same time in previous studies was difficult and time consuming). These results indicate that the infiltration function estimated using the suggested method can address errors in measurements (particularly in recession times) and improve the accuracy of the estimated infiltration depth. In addition, estimating the k parameter of the Kostiakov equation using Eq (11) can provide clear information about the variation of this parameter (k) along the furrow length. However, testing of the suggested method at another location with a different soil type and climate conditions is still required to validate this model.

## 4. Conclusions

In practice, the most common methods for estimating the infiltration function are the DRI method and the use of empirical infiltration models. The required data for infiltration determination always exhibit different kinds of scatter, which affect the accuracy of the estimated infiltration function.

This study suggests a new approach to reduce the effect of data scatter on the estimated infiltration function. The approach depends on the estimation of the infiltration function using a combination of a DRI and soil water content measurements.

First, the *α* parameter was obtained from a DRI experiment, and the *k* parameter was estimated using the proposed formula (Eq (12)), where the *α* parameter was equal to the value obtained from the DRI, and the opportunity times were obtained during the irrigation events at the same locations of the soil water content observation.

The infiltration function established using the suggested described approach was more accurate than that established using the other methods (the DRI and Kostiakov equation). The recommended approach was very useful for reducing the requirement measurements during the irrigation event, and therefore, reducing the effect of measurement errors on the estimated parameters, particularly when the observed recession times were not accurate, which is a common situation under field conditions in large-scale projects. In addition, estimating the *α* parameter from the DRI can help reduce the number of unknown parameters for solving the PIVB method. In this case, only the roughness coefficient (*n*), instead of both *α* and *n*, needs to be established with the PIVB method and through trial and error (with the initial value of *n* set, the *k* parameter is calculated using the PIVB method; this procedure is repeated until a good fit is achieved between the advance and recession times).

This study used the Kostiakov equation as an empirical model for estimating the infiltration function. However, using another empirical model (i.e., the modified Kostiakov model, which includes more empirical parameters) increases the accuracy of the estimated infiltration model. Additionally, the changes in the soil water content were observed at three locations along the furrow length. However, observing the soil water content at more locations increases the accuracy of the k parameter. Large variations in soil texture occur in the field; therefore, the soil texture in more locations in the study area should be determined for future studies. Furthermore, testing of the proposed method at another location with a different soil type and climate conditions is still required to validate this model.

## Supporting information

S1 File Measured (dots) and simulated (lines) advance and recession curves, inflow rate, and average inflow rate of furrow irrigation experiments. (DOCX)

S2 File Cumulative infiltration obtained from double-ring infiltrometer (DRI), Kostiakov equation (KE), and suggested approach of furrow irrigation experiments. (DOCX)

S3 File Infiltration rate obtained from double-ring infiltrometer (DRI), Kostiakov equation (KE), and suggested approach of furrow irrigation experiments. (DOCX)

S1 Table Observed relative elevations and calculated furrow slopes. (XLS)

S2 Table Inflow rate of furrow irrigation experiments. (XLSX)
