# Supplementary material for: An improved approach to estimating the infiltration characteristics in surface irrigation systems
Source: PLoS One. 2020 Jun 15;15(6):e0234480. doi: 10.1371/journal.pone.0234480 (PMC7295228; doi:10.1371/journal.pone.0234480)
Supplement: S3 File — (DOCX) [file pone.0234480.s003.docx]

|  |
| --- |
|  |
|  |
|  |
|  |
|  |
|  |
|  |
|  |
|  |
| **Figure S2**. Infiltration rate obtained from double-ring infiltrometer (DRI), Kostiakov equation (KE), and suggested approach of (a) furrow 1 ,(b) furrow 2, (c) furrow 3, (d) furrow 4 ,(e) furrow 5, and (f) furrow 6, (g) furrow 7, (h) furrow 8, and (i) furrow 9, (j) furrow 10, and (k) furrow 11. |
